# Validation of reference genes for gene expression studies in bovine oocytes and cumulus cells derived from *in vitro* maturation

**DOI:** 10.21451/1984-3143-AR2018-0064

**Published:** 2019-10-24

**Authors:** Lisandra Cristina Caetano, Cristiana Libardi Miranda-Furtado, Luciene Aparecida Batista, Caroline Palmieri Pitangui-Molina, Thaís Tiemi Higa, Cristiana Carolina Padovan, Ana Carolina Japur de Sá Rosa-e-Silva

**Affiliations:** Department of Gynecology and Obstetrics, Ribeirão Preto Medical School, University of São Paulo, Ribeirão Preto, SP, Brazil.

**Keywords:** cumulus cell, oocyte, reference genes

## Abstract

Quantitative real-time PCR (qPCR) is a valuable tool for gene expression studies and it is necessary to choose an ideal endogenous reference gene for data normalization. This work studied a set of reference genes in oocytes and cumulus cells of COCs (Cumulus-Oocyte Complexes) that are suitable for relative gene expression analyses after *in vitro* maturation (IVM) in bovine. Immature COCs were collected from ovaries of Nelore cattle (*Bos indicus*) and submitted to IVM. MII oocytes and cumulus cells were subjected to RNA extraction, reverse transcription and preamplification of cDNA. The expression level of eight reference genes (*ACTB, GADPH, B2M, H2AFZ, GUSB, HPRT1, PPIA, and TBP)* was measured by real time PCR and analyzed by geNorm software. The gene stability measure (M) was calculated and the ideal number of reference genes (RGs) was determined by the V value (pairwise variation). For oocyte samples, two RGs were the ideal number for relative quantification: *HPRT1* and *B2M* and for bovine cumulus samples four were indicated: *HPRT1, PPIA, B2M,* and *TBP* genes. The normalization of a non-reference target gene (*SOD1*) by these reference genes was shown to be considerably different from normalization by less stable reference genes. Our results strengthen the importance of choosing good normalizing genes in order to analyze gene expression under specific experimental conditions and we suggest the use of these RGs in oocytes and cumulus cells of bovine cattle in *in vitro* matured COCs.

## Introduction

Bovine cattle have a biological and economic importance in which reproductive traits are a relevant component of livestock and genetic breeding (Cammack *et al*., 2009). Bovine ovary physiology and some aspects of embryo growth and development are very similar to humans, which makes this species a possible translational model for developmental studies and improvement of assisted reproduction technologies (ARTs) in humans (Ross *et al*., 2009).

During follicular development, many mRNAs and proteins are synthetized, which regulate oocyte growth and maintain early embryonic development until the embryonic genome is activated (Gosden 2002; Sutton *et al*., 2003). In this period, the bi-directional interaction between oocyte cumulus and granulosa cells, also called crosstalk, is essential to complete oocyte development (Senbon *et al*., 2003). Granulosa cells intercommunication, as well as their interaction with the oocyte, occurs through gap junctions, where many substances like glucose, proteins, and mRNA pass through (Bruzzone *et al*., 1996; Sugiura *et al*., 2005; Gilchrist *et al*., 2008). As a result of this crosstalk, cumulus cells may reveal information about oocyte health through gene expression and many studies investigate these substances as noninvasive molecular markers for good quality oocyte selection (Sugiura *et al*., 2005; Gilchrist *et al*., 2008; Hamel *et al*., 2008).

The level of gene expression is frequently evaluated by reverse transcription (RT) associated with the quantitative polymerase chain reaction (qPCR). Values obtained are calculated using comparative analysis with respect to reference genes (RG), also known as normalizing genes or endogenous controls, which allow the removal of typical technical and biological variations (Livak and Schmittgen, 2001). Optimal RGs should be sufficiently abundant across different tissues and cell types, and have consistent RNA transcription under different experimental conditions. However, there is no universal reference gene for all cell types and experiments since gene expression is tissue, space, and time specific (Radonic *et al*., 2004). Thus, it is necessary to choose specific candidate genes for each experiment among three or more reference genes (Hruz *et al*., 2011). Specific algorithms were created to evaluate expression stability among different genes and define an optimal reference gene (or genes) for specific cell lines in RT-qPCR experiments. geNorm, NormFinder, and BestKeeper are some algorithms often used as analysis programs to validate RGs (Vandesompele *et al*., 2002; Pfaffl *et al*., 2004; Andersen *et al*., 2004).

Considering the importance of gene expression analysis for developmental studies and the use of bovine as a model to study aspects of assisted reproduction, we verified the absence of validation of reference genes in bovine samples submitted to *in vitro* maturation. Therefore, the aim this study was to evaluate the stability of eight candidates for reference gene in *in vitro* matured oocytes and cumulus cells from Nelore cattle (*Bos indicus*), analyzed by RT-qPCR.

## Methods

### Ethics statement

The present study was approved by the Bioethics Commission on Animal Experiments of the Ribeirão Preto Medical School, University of São Paulo (protocol number 073/2012), which complies with the ethical principles of animal research.

### Sample collection and in vitro maturation

Ovaries were transported from a local slaughterhouse to the laboratory in 0.9% saline solution supplemented with 0.05 g/L streptomycin at 35 to 37ºC and washed with 0.9% saline solution containing penicillin and streptomycin. Viable follicles measuring between 3 and 8 mm in diameter were aspirated with a 21-gauge needle adapted to a 20 mL syringe. Subsequently, COCs (Cumulus-oocyte complexes) were recovered from the follicular fluid of ovaries, washed in TALP-HEPES medium and evaluated for morphology under a stereomicroscope. Oocytes with a homogeneous, granulated cytoplasm and sufficient surrounding cumulus cells (with three layers or more), classified as category I or II were selected (Marquant-Le Guienne, 1999). Pools of ten immature COCs from the same cow were randomly assigned to *in vitro* maturation (IVM) in HEPES-buffered tissue culture medium-199 (TCM-199, Gibco/BRL, Grand Island, NY, USA) supplemented with 10% fetal calf serum (FCS, Invitrogen Gibco/BRL), 0.2 M sodium pyruvate, 100 IU/mL penicillin G, 100 μg/mL streptomycin, 0.5 μg/mL follicle stimulating hormone (FSH, Folltropin-Bioniche, Canada), 5 μg/mL luteinizing hormone (LH, Lutropin-Bioniche, Canada) and 1 μg/mL 17β-estradiol.

Maturation was carried out over a period of 24 h, at 38.5ºC, in an atmosphere with 5% CO_2_. Post-IVM, cumulus cells were mechanically removed from three COCs and stored together, in a total of 14 pools of cells. Oocytes were denuded in maturation medium supplemented with 0,2% hyaluronidase (Sigma-Aldrich, United States), under stereomicroscope. The oocytes presenting extrusion of the first polar body were washed in TCM medium with HEPES and stored in pool of four oocytes in micro tubes containing RNA*later* ^®^/PBS (1:3) for further analysis.

### Quantification of mRNA

Total RNA was extracted from the samples using the *RNeasy^®^ Micro kit* (Qiagen, Valencia, California) and all samples were treated with the *RNase-Free DNase Set* (Qiagen, Valencia, California) to digest all remaining DNA. RNA was reverse transcribed into cDNA with the *High-Capacity cDNA Reverse Transcription kit* (Applied Biosystems, Foster City, California) according to the manufacturer’s protocol. RNA concentration and purity were determined with a NanoDrop ND-2000 spectrophotometer, and samples with a 260/230 ratio higher than 2.0 and 260/280 ratio between 1.8 and 2.1 were selected and used in the subsequent experiment. Due to a low initial amount of cDNA we preamplified the material using the *Taqman PreAmp Master Mix kit* (Applied Biosystems, Foster City, California). After preliminary testing, the original protocol was modified to our laboratory conditions: 1X of Taqman *PreAmp Master Mix*, 0.05X of each assay in a pooled assay mix (0.2X), and 62.5ng of cDNA, in a 20-µl reaction volume. Preamplification cycles were: 95°C for 15min and 20 cycles of 95°C for 15sec and 60°C for 4min. The resulting material was diluted 20X and stored at 20°C pending RT-qPCR. According to the manufacturer’s instructions, we checked preamplification uniformity using non-limiting cDNA (extracted from ovarian tissue), prepared in the same way as the other samples.

Quantification of gene transcripts was carried out with the reverse transcription real-time polymerase chain reaction (RT-qPCR) using *TaqMan^®^ Gene Expression Assays* (Applied Biosystems, Foster City, California) available on the ThermoFisher website (http://www.thermofisher.com). The gene expression stability of optimal reference genes in oocytes derived from IVM was not analyzed in Nelore cattle by other authors. Among the reference genes available, eight were chosen for quantification in oocytes and cumulus cells, as follows: *ACTB, GADPH, B2M, H2AFZ, GUSB, HPRT1, PPIA, and TBP* (Table 1). Standard curves were generated by RT-qPCR using five 2-fold serial dilutions of preamplified cDNAs as a template to test for assay efficiency (data not shown). RT-qPCR analysis was performed using the Viia-7 system (Applied Biosystems, Foster City, California), in 10-µl reactions containing: 1X *TaqMan assay*, 1X *TaqMan Gene Expression Master Mix* (Applied Biosystems, Foster City, California), and 2.5 µl of template (preamplified sample diluted 20X). The mean value of the quantification cycle (Cq) was calculated from three technical repetitions per sample. The following cycling conditions were applied for all real-time RT-PCR reactions: initial denaturation at 95°C for 15min followed by 40 cycles consisting of 95°C for 15sec and 60°C for 1min.

**Table 1:** Reference genes to analyze expression stability.

Gene	Nomenclature	Function	Genebank acess	Taqman assay®
*ACTB*	actin, beta	Cytoskeletal structural protein	NM_173979.3	Bt03279174_g1
*B2M*	beta-2-microglobulin	Beta-chain of major histocompatibility complex class I molecules	NM_173893.3	Bt03251628_m1
*GAPDH*	glyceraldehyde-3-phosphate dehydrogenase	Oxidoreductase in glycolysis and gluconeogenesis	NM_001034034.2	Bt03210913_g1
*GUSB*	glucuronidase, beta	Carbohydrate metabolic process	NM_001083436.1	Bt03256165_m1
*H2AFZ*	H2A histone family, member Z	RNA polymerase II core promoter sequence-specific DNA binding	NM_174809.2	Bt03216348_g1
*HPRT1*	hypoxanthine phosphoribosyltransferase 1	Purine synthesis in salvage pathway	NM_001034035.2	Bt032225311_g1
*PPIA*	peptidylprolyl isomerase A (cyclophilin A)	Peptidyl-prolyl cis-trans isomerase activity	NM_178320.2	Bt03224615_g1
*TBP*	TATA box binding protein	General RNA polymerase II in transcription factor	NM_001075742.1	Bt03241946_m1

### Stability analysis

Gene expression stability was analyzed using qBasePLUS software v.2.3 (Biogazelle, Zwijnaarde, Belgium) based on the geNorm algorithm (Vandesompele *et al*., 2002). The algorithm hypothesizes that the expression ratio of two ideal reference genes should be identical in all samples, independently of cell type or experimental design. According to Vandesompele *et al*., (2002), the software determines the gene-stability measure (M) of a given reference gene using an algorithm based on the mean pairwise variation compared to all other control genes. Larger M values are associated with greater variations in gene expression. geNorm computes the optimal number of reference genes required for accurate normalization by calculating Vn/n+1 pairwise variation between consecutively ranked normalization factors NFn and NFn+1, where n and n+1 are the number of genes considered, and NFi are the geometric means of the *i* best candidate reference gene transformed Cq values. A pairwise variation of 0.15 is suggested as a cut-off value, below which the inclusion of an additional reference gene is not required for reliable normalization (Vandesompele *et al*., 2002).

### Determination of the SOD1 expression profile

To understand the relevance of reference gene selection for gene expression experiments, a non-reference target gene (*SOD1*) was used and normalized to different RGs. S*OD1,* also known as superoxide dismutase 1, is an integrant of the enzymatic antioxidant system related to the balance of free radicals and is expressed in both oocyte and cumulus cells (Cetica *et al*., 2001). The cDNA for this gene was preamplified and tested with all samples, oocytes and cumulus cells (TaqMan assay Bt03215424_g1 - Applied Biosystems, Foster City, California). Since all real-time RT-PCR assays showed high amplification efficiency, target transcript amounts in each sample were linearized according to Livak and Schmittgen (2001) by 2^-ΔΔCq^ (normalized values) to analyze SOD1 gene expression levels.

## Results

### Expression profiles of candidate RGs and geNorm analysis of stability

**Figure 1:**
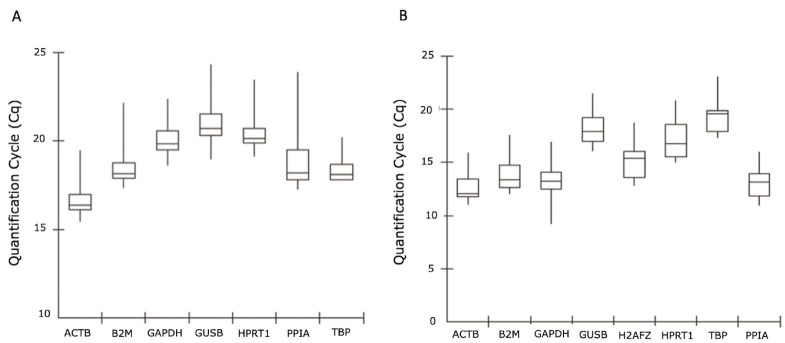
Quantification cycle (Cq) values of candidate RGs across the experimental samples (A) oocyte and (B) cumulus cells. A box-plot graph of Cq values shows the median values as lines across the box. Upper and lower boxes indicate the 75^th^ percentile to the 25^th^ percentile. Whiskers represent the maximum and minimum values.

In order to define the best RGs for bovine *in vitro* matured oocytes and cumulus cells, eight genes were selected for qPCR analysis according to their frequency in the previous literature: *ACTB, GADPH, B2M, H2AFZ, GUSB, HPRT1, PPIA, and TBP*. The expression profile of candidate RGs, represented as mean Cq values, were used to determine the most adequate gene for comparative quantification. The amplification data showed a range of Cq values from 15 to 24 for oocytes and from 9 to 23 for cumulus cells (Figure 1).

**Figure 2:**
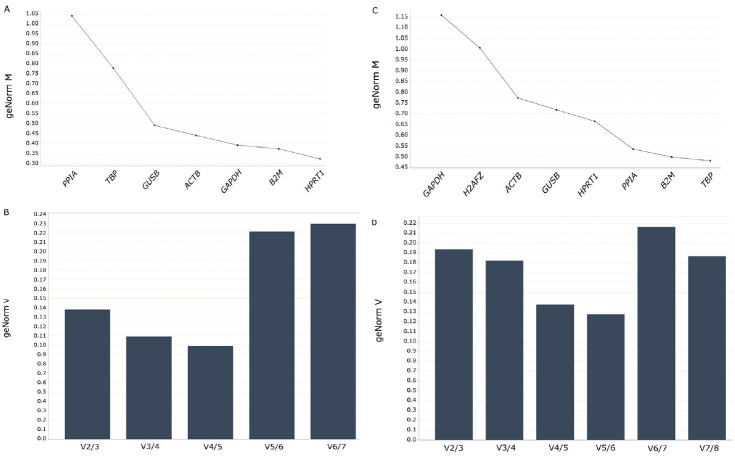
Average expression stability values (geNorm M) of evaluated genes in oocyte (A) and cumulus cells (C) from Nelore cattle, plotted from least stable (left) to most stable gene (right). Graphics B and D shows the gene expression pairwise variation (V) of the candidate RGs calculated by geNorm. Values smaller than 0.15 are considered acceptable as the optimum number of RGs [13].

The geNorm algorithm revealed gene expression stability among analyzed RGs in oocytes and cumulus cells (Figure 2). An optimal geNorm experiment contains at least 10 representative samples and eight candidate reference targets (Vandesompele et al., 2002). We analyzed eight reference genes in 14 samples of oocytes and cumulus cells. One sample of oocytes contained missing data and was excluded from the analysis. The *H2AFZ* showed no amplification for oocytes and was excluded from the software analysis.

When all oocytes samples were examined together, the average expression stability value (M) was highest for *PPIA* and lowest and more stable for *HPRT1* and *B2M* (Figure 2A). The optimal number of reference targets in this experimental situation is two genes to normalize qPCR results (geNorm V < 0.15 when comparing a normalization factor based on the 2^nd^ or 3^rd^ most stable targets) (Figure 2B). Thus, the optimal normalization factor for oocytes can be calculated as the geometric mean of reference targets *B2M* and *HPRT1. GAPDH* and *ACTB* genes, commonly used as RGs, showed less variability between samples when compared to *TBP* and *PPIA,* and the most unstable reference gene for expression analysis being the *PPIA* for IVM oocytes.

The analysis of 14 cumulus samples revealed higher values of M for *GAPDH* than the other reference genes, which includes the GAPDH as the more unstable gene for IVM cumulus cells gene expression studies (Figure 2C). On the other hand, the genes *HPRT1, PPIA, B2M,* and *TBP* seem to be more stable for cumulus cell gene expression. The optimal number of reference targets in this experimental situation is 4 (geNorm V < 0.15 when comparing a normalization factor based on the 4^th^ or 5^th^ most stable targets). This way, the optimal normalization factor can be calculated as the geometric mean of reference targets *HPRT1, PPIA, B2M, and TBP* (Figure 2D).

### RG validation by quantification of the SOD1 expression profile with different normalization factors

To validate whether the most stable genes selected by the geNorm program were the most suitable to quantify for normalization, different RGs were used for normalization of *SOD1* expression. As observed in Table 2, unstable RGs lead to alterations in relative gene expression values of *SOD1* in both oocytes and cumulus cells, varying between under- and/or over-expression results, while the selected RGs showed good stability and lower variation.

**Table 2:** Relative quantification of SOD1 gene using the different RGs or a combination according geNorm analysis.

Group	Sample	ACTB	B2M	GAPDH	GUSB	H2AFZ	HPRT1	PPIA	TBP	geNorm RGs
	118A	1,02	1,23	1,58	1,35	1,64	0,91	1,87	1,19	1,26
	120A	1,32	0,98	2,88	2,47	1,86	1,01	1,18	1,33	1,12
	121A	1,13	1,13	0,85	1,39	1,77	1,14	1,46	1,24	1,23
	122A	0,93	1,57	1,66	1,98	2,64	1,55	1,61	1,92	1,66
	123A	0,58	1,38	0,15	2,27	0,85	1,75	1,84	0,92	1,42
CC	127A	0,80	0,92	0,92	1,41	1,47	0,77	1,82	0,89	1,03
	128A	1,08	0,81	1,33	3,43	1,38	0,94	0,84	1,17	0,93
	129A	0,86	0,52	1,22	2,55	1,07	1,17	1,44	0,57	0,84
	146A	0,66	0,47	0,08	2,48	0,20	1,48	0,55	0,48	0,66
	151A	0,49	0,31	0,73	2,25	3,15	1,74	1,31	0,65	0,82
	157A	0,25	1,05	0,76	1,40	1,69	1,94	1,41	0,96	1,29
	159A	0,53	0,94	1,24	1,80	2,21	2,34	1,81	0,94	1,39
	161A	0,20	0,50	0,20	1,66	0,26	1,64	0,82	0,48	0,75
	164A	0,64	0,59	13,09	1,27	28,53	1,47	2,00	0,85	1,10
										
	121	0,60	0,51	0,50	0,54	-	0,73	0,43	0,54	0,61
	122	0,76	1,01	0,95	1,49	-	1,01	0,93	1,49	1,01
	123	1,52	1,28	1,08	1,67	-	1,01	0,59	1,67	1,13
	125	2,17	2,25	1,95	1,63	-	2,09	0,91	1,63	2,17
	129	1,08	1,51	1,02	0,64	-	1,29	0,95	0,64	1,40
	132	1,64	1,26	0,69	0,64	-	1,02	0,44	0,64	1,13
O	135	0,70	1,67	0,56	1,05	-	1,04	0,31	1,05	1,32
	145	1,68	1,76	1,46	1,33	-	1,58	1,03	1,33	1,67
	162	0,81	1,92	2,01	1,06	-	1,88	2,11	1,06	1,90
	173	1,42	1,70	1,26	0,74	-	1,46	0,94	0,74	1,58
	176	0,59	0,69	1,10	0,70	-	0,83	0,72	0,70	0,82
	181	1,04	1,86	1,26	1,20	-	0,84	49,90	1,20	1,65
	192	0,77	1,23	1,33	0,81	-	1,14	0,89	0,81	1,19

CC- cumulus cell samples, O- oocyte samples, and geNorm optimum RGs analyzing cumulus cell samples were: *HPRT1, PPIA, B2M,* and *TBP*, and oocytes were: *HPRT1* and *B2M*. H2AFZ did not avalaible in oocytes.

## Discussion

In this study the stability of *ACTB, GADPH, B2M, H2AFZ, GUSB, HPRT1, PPIA, and TBP* as reference genes was analyzed in order to select the more adequate one to be used in gene expression studies of bovine oocytes and cumulus cells derived from *in vitro* maturation. We observed that the best RGs for oocytes gene expression studies were the *B2M* and *HPRT1* genes. On the other hand, the *HPRT1, PPIA, B2M,* and *TBP* seem to be more stables for cumulus cell sample.

The increasing advancement of molecular biological techniques allows the investigation of gene expression patterns at the transcript level (Hamel *et al*., 2008; Ross *et al*., 2009; Salhab *et al*., 2013). RT-qPCR is a frequently used technology for specific gene expression evaluation and comparative analysis of stable RGs determined for each specific cell type increases reliability (Vandesompele *et al*., 2002; Radonic *et al*., 2004; Pfaffl *et al*., 2004; Andersen *et al*., 2004). *ACTB, GAPDH, H2AFZ, H2A, HPRT1, PPIA,* and *RNA18S* are the most common genes used in such studies involving the bovine model (Patel *et al*., 2007; Biase *et al*., 2009; Macabelli *et al*., 2014). In other mammals, especially human, sheep, and mouse, different reference genes are more common, such as *TBP, GUSB,* and *B2M* (Ragni *et al*., 2013).

Although many studies have analyzed gene expression in oocytes and cumulus cells, there was no concordance regarding the ideal combination of RGs for these specific samples, and validation for reference genes is not commonly applied for small samples such as oocytes. However, according to the current consensus is necessary to evaluate the expression stability of RGs for a specific experimental condition (Livak and Schmittgen 2001; Radonic *et al*., 2004; Dheda *et al*., 2005).

Macabelli *et al*., (2014) published their data regarding validation of reference genes for *in vivo* matured oocytes from Holstein cattle (*Bos taurus*) and buffalo (*Bubalus bubalis*), analyzing the effect of summer on fertility. They studied *ACTB, GAPDH, GUSB, HIST1H2AG, HPRT1, PPIA, RPL15, SDHA, TBP, and YWHAZ* reference genes and concluded that owing to the high variability of reference genes among their bovine experimental groups, data normalized by internal controls could be misunderstood. They suggested that ideal experiment should include comparisons to non-normalized data or data normalized by an external control, in order to better interpret the biological relevance of gene expression analysis (Macabelli *et al*., 2014).

In another study, regarding gene expression and maternal aging, reference genes (*GAPDH*, *ACTB, UBE2D2*, *EIF2B2*, *SF3A1*, *RNF20*) of cumulus cells from dominant follicles in cows (an unrelated bovine breed) were analyzed (Khan *et al*., 2016). They observed that the geometric mean of multiple genes (*UBE2D2*, *EIF2B2*, *GAPDH,* and *SF3A1*) is a more appropriate reference control than the use of a single reference gene to compare relative gene expression among dominant and FSH-stimulated follicles during maternal and/or follicular aging studies. However, Khan and collaborators (2016) showed that RGs for cumulus cells had a higher variability, may be due to a large number of cells and developmental stages of each cell of the specific COCs. In humans, one study showed that the best RGs for human cumulus cells are *UBC* and *B2M* (Adriaenssens *et al*., 2010).

Analyzing different RGs for normalization of *SOD1* gene expression, unstable RGs lead to altered gene expression values of *SOD1* in both oocytes and cumulus cells. We can observe that greater genetic instability leads to greater variation in gene expression, which may overestimate or underestimate the data, introducing erroneous results (Andersen *et al*., 2004; Radonic *et al*., 2004; Hruz *et al*., 2011; Macabelli *et al*., 2014). So the validation of the greatest RGs for each gene expression studies using qPCR is mandatory to have more accurate and robust results, with high specificity, eliminating the bias of qPCR studies. Although we analyzed *SOD1* gene expression, in this study we did not evaluate any cumulus specific gene to exclude contamination in oocytes samples by eventual remaining cumulus cells. However, the cumulus cell were carefully removed under stereomicroscope, and the oocytes cells which were not totally cleaned were excluded from this study.

Gene expression studies are great tool to understand and estimate cell stage and physiological responses to environmental alterations. The primary step of these studies is to choose an adequate gene to normalize the expression of a target gene, reducing technical and biological variations. In this context, according to the geNorm algorithm, we suggest *HPRT1* and *B2M* to be the best RGs for bovine *in vitro* matured oocytes and *HPRT1, PPIA, B2M,* and *TBP* to be the best normalizers for cumulus cells. To the best of our knowledge, this is the first study validating reference genes in *in vitro* matured oocytes of Nelore cattle, an important species from an economic standpoint and from a biological point of view for animal breeding programs.
